# Central Nervous System Impact of Perinatally Acquired HIV in Adolescents and Adults: an Update

**DOI:** 10.1007/s11904-021-00598-3

**Published:** 2022-02-02

**Authors:** Sharon L. Nichols

**Affiliations:** Department of Neurosciences, University of California, San Diego 9500 Gilman Drive, #0935, CA 92093 La Jolla, USA

**Keywords:** HIV, Perinatal, Neurocognitive, Neuroimaging, Adolescent, Young Adult, Youth

## Abstract

**Purpose of Review:**

Perinatally acquired HIV infection (PHIV) can confer neurodevelopmental risk. As children with PHIV increasingly survive through adolescence and into adulthood, understanding its long-term central nervous system (CNS) impacts is critical for maximizing adult outcomes and quality of life.

**Recent Findings:**

Recently published neurocognitive and neuroimaging findings show impacts on the CNS associated with early HIV disease progression that endure into adolescence and young adulthood. Although developmental trajectories in adolescence largely appear stable, further research on maturational processes is indicated.

**Summary:**

Although early antiretroviral therapy in infancy appears to be protective, it is not universally available and current youth largely developed without its benefit. The neurocognitive effects of HIV and the multiple other risks to neurodevelopment experienced by youth with PHIV call for further longitudinal research and a multifaceted approach to prevention and intervention.

## Introduction

Despite our ability to prevent mother to child transmission of HIV through administration of antiretroviral therapy (ART) during pregnancy, birth, and breastfeeding, pediatric HIV continues to be a global concern, with 150,000 new pediatric infections worldwide in 2019 [1]. Furthermore, effective ART has enabled those children with perinatally acquired HIV (PHIV) who have access to treatment to survive through childhood and adolescence. However, globally, ART access for children under age 15 (53%) lags behind that for adults [2] and HIV remains one of the leading causes of death for adolescents [3, 4]. As growing numbers of youth with PHIV enter adulthood, the long-term impact of living with HIV throughout development on the health and daily life of these young adults, and the potential benefits of ART for quality of life as well as survival, take on greater importance. The purpose of this review is to highlight recent findings regarding the effect of PHIV on a key area of concern, central nervous system (CNS) functioning and structure, during adolescence and young adulthood.

That HIV can have significant neurologic and neurocognitive effects has been well known since the early years of the epidemic, and prior to the introduction of ART, particularly combination ART (cART) in 1995, perinatal acquisition was associated with a high risk for poor brain development, encephalopathy and other neurologic sequelae [5]. Since then, the picture of CNS complications of PHIV has evolved as therapies have been refined, availability has increased, albeit at different rates in various regions of the world, and guidelines regarding treatment initiation have changed. At the same time, studies conducted largely in adults have described ongoing impacts of HIV on the CNS despite ART and clarified many aspects of neuropathogenesis; these include early HIV entry into the CNS, ongoing effects through immune activation and inflammation despite viral suppression, maintenance of a viral reservoir in the CNS compartment, and varying neurotoxicity of ART regimens [6, 7]. In both youth and adults, critical issues include HIV’s evolving and often subtle functional effects, disentangling them from other influences on CNS functioning, impact on everyday functioning, and development of preventive and rehabilitative therapies.

A number of recent reviews have summarized the literature on cognitive and neuroimaging findings among children with PHIV [5, 8–15]. In general, these reviews acknowledge that, despite decreases in severe neurological sequelae since the advent of cART for children, cognitive impairments continue to be a concern, particularly in the developing world where ART is less available, older antiretroviral medications with greater neurotoxicity are more commonly used, and HIV encephalopathy continues to occur [16]. Systematic reviews note that decreased functioning is apparent in specific domains of cognitive functioning, such as executive functioning (particularly working memory), processing speed, visual memory, and visuospatial ability [8, 11]. However, the degree to which impairments are attributed to HIV versus other developmental, environmental, and socioeconomic risks varies. Young people with PHIV are disproportionately subject to environmental and psychosocial stressors and societal inequities that have potential impact upon development. Recognition of the role of these other risk factors and the inappropriateness of test standardization for many contexts calls for carefully matched comparison groups. These typically consist of children with perinatal HIV exposure but uninfected (PHEU), and/or, in recognition of possible effects of HIV exposure itself [17–19], unexposed children matched to those with PHIV on demographic and socioeconomic characteristics hypothesized to play an important role in neurodevelopmental outcomes.

Across the pediatric HIV literature, a key predictor of altered neurodevelopment has been history of HIV disease severity, generally with stronger associations than current immune functioning or viral activity. Significantly lower functioning is often reported among children with PHIV and a history of CDC Class C, or AIDS, diagnosis [20], particularly when it includes diagnosis of encephalopathy [5, 21, 22]. A related finding is the neurodevelopmental benefit of earlier initiation of cART through preventing significant HIV activity and disease during crucial periods of development [23, 24]. Studies such as the Children with HIV Early Antiretroviral Therapy study in South Africa have suggested that long-term neurodevelopmental impacts of HIV, resistant to later disease control, begin in the first few months of life [25]. These, as well as other findings, have contributed to current guidelines that call for initiation of ART during infancy or immediately upon diagnosis of HIV during childhood [26].

Given this, it is clear that research on children more recently born with PHIV cannot easily be extrapolated to those who are currently adolescents or, particularly, young adults who progressed through neurodevelopment in an earlier era of HIV treatment. Although there is wide variability across countries, current adolescents with PHIV in lower-income settings such as sub-Saharan Africa initiated ART at a median of almost 8 years of age [27] and have impacts of HIV on multiple systems, including the CNS [28, 29]. Similarly, it is reasonable to assume that the effects of HIV present throughout postnatal, and in some cases, prenatal, brain development would differ from those of HIV acquired during adolescence or adulthood. Thus, our understanding of adolescents and young adults currently living with PHIV requires targeted studies.

This research remains relevant despite significant changes in treatment strategies for two reasons. First, youth with PHIV have the prospect of long lives, accompanied by the opportunities and responsibilities faced by any young adult. By addressing the unique experience and effects of PHIV for youth, scientists and clinicians can prepare and assist them in their transition to adulthood and maximize their long-term health and quality of life. Neurocognitive functioning has been linked with a variety of daily functioning, educational/occupational, and behavioral outcomes (including risk behaviors that increase the likelihood of negative health outcomes) among children with PHIV [30–35]. However, its influence during the critical and risky period of transition to independent adulthood and responsibility for their own healthcare among those with PHIV is not yet well understood [36].

Second, despite guidelines for initiating ART during infancy for those with PHIV, there continue to be significant numbers of children who do not receive early ART, with only about half of children with PHIV in sub-Saharan Africa having access to ART in 2019 [37]. A far lower percentage initiate treatment by age one [12]. Thus, many children continue to be exposed to the risk of long-term CNS complications. Unfortunately, the world does not yet have in sight an end to our need to understand and assist this population.

The goal of this article is to highlight selected recent (since 2015) articles on CNS functioning and integrity among adolescents and young adults with PHIV, primarily studies focused on youth age 10 and up to reflect current definitions of adolescence [38]. Relevant articles use neurocognitive measures or neuroimaging paradigms to evaluate the state of the CNS; papers addressing mechanisms and biomarkers of neuropathogenesis are outside the scope of this review. My hope is to leave the reader with an understanding of the current state of the literature and a recognition of its clinical relevance.

## Neurocognitive Functioning in Youth with PHIV

### Adolescents

Several longitudinal cohort studies of children and youth with PHIV, ongoing on several continents, have in recent years published findings regarding neurocognitive functioning in participants as they reach and proceed through adolescence. Among the larger cohort studies now following adolescents are the PREDICT study of the impact of early versus deferred ART initiation conducted in Thailand and Cambodia [24, 39, 40] and the Pediatric HIV/AIDS Cohort Study (PHACS) of long-term effects of PHIV infection and perinatal HIV and ART exposure in the USA (https://phacsstudy.org/About-Us) [41].

The RESILIENCE substudy of PREDICT examined long-term cognitive and behavioral functioning of 231 youth with PHIV age 10-24 and age- and sex-matched youth with (HEU; *n*=125) and without (HUU; *n*=138) PHIV exposure [42•]. Assessments at baseline and three annual follow-up visits focused on executive functioning, memory, processing speed, and internalizing and externalizing behavioral problems. To address lack of locally standardized tests, impairment was defined according to the HUU mean. Youth with PHIV, compared to HUU, had higher risk of impairment in executive functioning and visual memory, lower mean scores on processing speed and working memory, and reported more internalizing and externalizing behavioral problems. Additional analyses with a subset of RESILIENCE participants [43] found higher caregiver-rated inattention, but not hyperactivity, among youth with PHIV. These findings are particularly noteworthy because of the low rate of past disease progression and Class C diagnoses among the PREDICT cohort, and their good viral control; nevertheless, most youth with PHIV had initiated cART after age 2 suggesting that is too late to avoid long-term impacts. However, the reported findings support some optimism as the highest rates of impairment observed were less than 20% for the group with PHIV, and mean standardized scores based on published data were in the low average to average range for most test scores.

The Memory and Executive Functioning substudy of PHACS assessed youth age 9–18 with PHIV and no Class C diagnosis (PHIV-NonC; *n*=105), PHIV and Class C diagnosis (PHIV-C; *n*=39), and PHEU (*n=*79). A series of publications [44–47] reported that, after adjustment for sociodemographic variables, adolescents with PHIV-NonC and PHEU were comparable on most measures. Those with PHIV-C performed significantly worse on visual recognition memory, some aspects of prospective memory, and inhibitory functioning. Effects on other areas of executive functioning were not observed, and previous analyses of the broader PHACS cohort suggested EF problems among youth with PHIV in the USA may be related to other risk factors [48]. There were few associations with current immunological or viral parameters, although a relationship between CD4 and verbal learning raised concerns about ongoing impact on this critical area of functioning. Comparison to publisher age standardization data was consistent with previous studies in showing mean performance in the average range overall but shifted downwards, and with a relatively higher proportion of scores in the impaired range across groups but particularly among those with PHIV-C. Associations with sociodemographic and education-related covariates support significant contributions of socioeconomic factors to neurodevelopment among youth with or exposed to HIV perinatally. Interestingly, associations with age at greatest disease severity did not always support greater impact with earlier disease progression. This may reflect differences in the periods of greatest vulnerability for later-maturing cognitive domains such as executive functions.

Most recently published analyses of adolescent neurocognitive functioning have been cross-sectional, but increasingly, change is being examined. A two-year follow-up of youth enrolled in the PHACS memory and executive functioning study described above showed stable functioning over time for all three groups [44]. It should be noted that those with PHIV largely maintained good immune health over the follow-up period, which may have been protective. The NOVICE study, conducted in the Netherlands [49], enrolled children with PHIV and community controls ages 8–18, conducting cognitive assessments at baseline and at follow-up an average of 4.6 years later when participants’ mean ages were 18 (PHIV; *n*=21) and 17 (HIV-; *n*=23). Although both groups were majority Black, those with PHIV were more likely to be born in sub-Saharan Africa and to be adopted or in foster care. Youth with PHIV had lower performance than those without on most measures, both groups performing lower than standardization samples. However, although change was similar on most measures, the performance of youth with PHIV on executive functioning tasks dropped over time despite good overall viral control, while that of the HIV- group remained stable. The authors interpreted the increasing difference in executive functioning over time according to the “growing into deficit” hypothesis, i.e. impact on executive functioning will become more apparent as youth enter a stage of rapid maturation of that domain. Thus, although stability in most cognitive domains in these studies is reassuring, the suggestion of a decline in executive functioning relative to age expectations among those with PHIV requires additional study, particularly in light of this domain’s importance to maintenance of health, acquisition of skills for transition to adulthood, and decision-making in behavioral risk contexts. In addition, their functioning remains depressed compared to societal age expectations, emphasizing the importance of preventive and rehabilitative interventions. A recent study including younger children, limited to PHIV, that used group-based trajectory analysis identified three distinct trajectories; risks for poor outcomes included older age at ART initiation and poverty [50].

### Young Adults

As more youth with PHIV have survived into adulthood, an increasing number of publications have presented findings regarding their neurocognitive functioning, largely in North America and Europe where effective ART first became widely available but in some cases including a large proportion of immigrants. The Adolescents and Adults Living with Perinatal HIV (AALPHI) study in England [51] enrolled 296 youth with PHIV (age 13–21), with and without history of Class C diagnosis, and 97 uninfected youth (age 13–23) related to or friends with a participant with PHIV between 2013 and 2015; thus, participants were born largely during the late 1990s. The majority of participants were Black, with a large proportion of immigrants and bilingual youth particularly among those with PHIV. Those with PHIV and class C diagnosis had lower performance than those with no Class C diagnosis and uninfected youth in executive functioning, speed of information processing, memory, and fine motor skills, while both groups with PHIV had lower scores in the learning domain than those without HIV. Also, consistent with other reports, all groups showed impaired functioning compared to standardization data, with the limitation, common to studies of PHIV, that publishers’ normative samples were poorly matched to the study population across demographic and socioeconomic variables. However, the authors note that impairment was generally mild, youth with PHIV and no history of severe disease were comparable to uninfected youth in almost all areas, and associations of lower performance with socioeconomic deprivation and depression suggest alternative, potentially modifiable, influences.

Robbins and colleagues [52•] enrolled 206 children with PHIV and 134 with PHEU in New York at ages 9 to 16 during the period 2003 to 2008. Tests of executive functioning, working memory, and processing speed were administered over four time points when the participants were ages 15–29 years. The performance of both groups was lower than standardization samples but generally comparable, particularly in the absence of AIDS-defining illness among those with PHIV. Longitudinal analyses showed improvement in most test scores over time with few differences between groups; however, a notable exception was improvement in processing speed among YPHEU that was not apparent among YPHIV. The authors note that the failure of the group with PHIV to improve in processing speed over time may represent a vulnerability of this group that signals risk for future cognitive problems. As research seeks predictors of negative long-term outcomes to aid prevention and intervention efforts, findings such as this provide important directions for future studies.

Other recently published studies of young adults are limited by small sample sizes or the lack of a matched comparison group. Willen and colleagues used MRI and a brief assessment of cognitive functioning focused on executive functioning to compare 29 young adults with PHIV, ages 18–24, to 13 healthy community controls in the USA [53]. They found worse functioning among the PHIV group across most tests but no consistent associations with HIV disease variables. Garcia-Navarro and colleagues evaluated intellectual functioning among 97 children through young adults with PHIV in Spain, 40% of whom were in the age range 17–23 years [54]. They found few differences between findings for older and younger participants; across both groups, standardized scores for crystallized intelligence were lower than for fluid intelligence. Although CDC classification, age at HIV diagnosis and cART initiation, and immunological parameters were associated with intellectual functioning, sociodemographic variables such as primary language and caregiver education played a significant role.

Coutifaris and colleagues took an interesting approach to examining the influence of age at HIV infection on neurobehavioral functioning [55]. In this study conducted in New York, they compared 13 each of young adults ages 20–30 with PHIV, age-matched young adults with behaviorally acquired HIV (BHIV), and older adults with duration of HIV infection and reading ability comparable to those with PHIV (mean age 56.6). All but one of those with PHIV had a history of AIDS diagnosis, along with 10 of the older adults, and the two groups were also similar in having been diagnosed with HIV before availability of cART. Their findings showed higher rates of cognitive impairment and psychiatric dysfunction (both 85%) among those with PHIV compared to both other groups. Those with PHIV performed significantly worse than the other groups in global cognition, speed of information processing, working memory, and verbal fluency. Adverse environmental factors were common among the PHIV group, and both young adult groups had about a third with positive urine toxicology for cannabis. The deficits among the group with PHIV could relate both to unique characteristics and vulnerability of the CNS during early development and to the participants’ histories of systemic HIV disease, together resulting in impairments that are refractory to later cART and immune reconstitution [56]. The impact of early systemic illness, as noted by the authors, can also extend beyond its biological impact on the brain to include lost developmental, social, and educational opportunities and contribution to the overall burden of adversity.

## Selected Recent Neuroimaging Studies Among Adolescents and Young Adults with PHIV

Several recent systematic reviews have summarized neuroimaging studies of children and adolescents with PHIV across both higher- and lower resource settings [9, 13, 14, 57]. Broad conclusions have been elusive due to differences in methodology, treatment status and history, timing of data collection relative to treatment guidelines, and range of other data collected such as cognitive functioning, medical history, and family and environmental variables. These reviews note both structural and functional findings with particular consistency regarding cortical thickness, white matter abnormalities and microstructural differences among children with PHIV. Since 2015, a number of neuroimaging studies focused specifically on adolescents and young adults with PHIV have described findings across a range of settings.

The majority of recent studies address brain structural findings by comparing youth with PHIV to age-matched youth without HIV infection [58–62] or to large normative databases [63, 64, 65•]. These studies have noted differences in white matter integrity [61, 66] and cortical and subcortical gray and white matter volume that are associated with performance on cognitive measures [58–60, 64] and markers of systemic inflammation [66], and differences in white matter microstructure using DTI [61]. Lewis and colleagues [64] compared a cohort of 40 US youth with PHIV (mean age 16.7) with 334 age-matched youth from the large NIH-funded Pediatric Imaging, Neurocognition, and Genetics (PING) study, showing lower total and regional gray matter volume that had significant associations with cognitive functioning and past and recent viral load. This was one of the only studies to examine substance use, an important confounding influence for adolescents, among the group with PHIV, demonstrating associations of smaller gray matter volumes with alcohol and marijuana use. This group has also reported associations of deformation of subcortical structures with markers of past disease severity and cognitive functioning among PHIV, suggesting this as a potential clinical marker [63]. In a Chinese cohort of 25 adolescents with PHIV and 33 uninfected but living with an HIV-infected parent (mean age 15 years), Li and colleagues [59] used voxel-based morphometry to show region-specific decreases in both gray and white matter volume and white matter density among those with PHIV. In addition, they presented associations of anterior cingulate cortex volume with cognitive performance and current CD4 cell counts, and associations of age of cART initiation with gray matter volume. Hypothesizing that structural brain networks would be affected by the presence of HIV during brain development and maturation, these investigators compared gray matter covariance networks and their organization between the two groups [58]. Structural network analyses showed increased centrality in frontal regions and decreased in temporal regions in adolescents with PHIV compared to uninfected adolescents, as well as shifted distribution of network hubs.

Several recent papers have focused on structural measures such as cortical thickness, surface area, and gyrification [62, 65•, 66] that have increasingly been used in studies of HIV among children [67], as well as adults [68–72], and are sensitive to developmental processes. The few studies focusing on adolescents and young adults show regionally specific decreases in cortical thickness, including temporal, orbitofrontal, and occipital lobes in combination with lower gray matter volume in subcortical structures among Spanish young adults [62], decreased cortical thickness in right temporal lobe and fusiform gyrus among Zambian adolescents with PHIV [73], and increased thickness in left occipital and right olfactory areas and decreased cortical thickness in temporal and orbitofrontal regions among Chinese youth [60]. Lewis and colleagues described decreased cortical thickness, surface area, and gyrification in frontal, parietal, and temporal regions among the US adolescents with PHIV compared to an age-matched PING cohort; the distribution of differences varied across the three metrics, which reflect different developmental processes [65•].

As of yet, few longitudinal neuroimaging studies have examined maturational changes during adolescence in the context of PHIV. Yu and colleagues followed 16 Chinese youth with PHIV and 25 without, age 11–17, for one year with repeat measurement of gray matter volume and cortical thickness along with cognitive testing [60]. Although both groups showed developmental cortical thinning and reductions in gray matter volume after one year, their topography differed, leading the authors to suggest delayed cortical maturation with PHIV. In contrast and in a younger cohort, the PREDICT study performed shape analysis of subcortical structures over two time points one year apart in an early adolescent cohort of Thai youth (mean age 11 at baseline) with PHIV, PHEU, or uninfected and unexposed [74]. Although there were group differences in the pallidum at baseline, these attenuated over one year and group effects were considered minor by the authors; however, within the group with PHIV intriguing associations of CD4 count with pallidum shape and volume were observed. Similarly, findings from the Dutch NOVICE cohort suggest comparable maturational processes during later adolescence [75]. Longitudinal structural MRI and diffusion tensor imaging (DTI) were performed over 4.6 years for 20 youth with and 23 without HIV (mean age 18 and 17, respectively, at the follow-up visit). Those with PHIV showed lower total white matter volume, lower fractional anisotropy, higher mean diffusivity and radial diffusivity. These differences were maintained over time, with both groups showing typical and comparable increases in white matter volume and decreases in gray matter volume. Thus, these longitudinal studies generally agree with neurocognitive studies showing typical developmental processes during adolescence, albeit with prior static differences preserved. However, intriguing cross-sectional examinations of age, or maturation, effects include Lewis and colleagues’ findings showing the typical inverse relationship of gray matter volume with age, thought to reflect maturational pruning, in the PING, but not the PHIV, group [65•]. This suggests atypical maturational processes during adolescence, at least among their cohort of youth who did not receive ART in infancy. Interestingly, in a slightly younger cohort of South African children age 9–12, Hoare et al. [76•] found that measures of cortical thickness, as well as surface area, volume, and neuronal microstructure, correlated more highly with epigenetic age than chronological age, suggesting epigenetic age acceleration in PHIV. This is significant in light of other evidence associating extrinsic epigenetic age acceleration with lower cognitive functioning in this cohort [77] and in a cohort of young African American adults with PHIV [78].

Few studies published since 2015 have examined functional connectivity and brain networks among adolescents to young adults with PHIV. In the only recent study to use resting state functional MRI (rs-fMRI) with young adults with PHIV, Sarma [79] showed higher regional homogeneity, a marker of local functional organization, and amplitude of low frequency fluctuations (ALFF) in white matter in the medial orbital gyrus among a small US sample of 11 youth with PHIV age 18–30 compared to uninfected youth. Almost half of the participants with PHIV had past definitive or probable diagnoses of HIV encephalopathy. Both measures were associated with viral load and with performance on several cognitive measures; the authors did not report associations with past disease severity, which may have been precluded by the small sample size. These authors interpret their findings as reflecting ongoing neuroinflammation and possibly glial cycling. Another small study conducted in China [80] similarly examined (ReHo) regional homogeneity among 13 treated adolescents age 12–15 with PHIV and 22 uninfected youth. Those with PHIV showed both areas with higher ReHo in central somatic motor-sensory cortex and lower ReHo in corticostriatal pathways previously seen as vulnerable to HIV. No associations of ReHo with a cognitive screening measure or nadir CD4+ T cell counts were seen. Finally, Heany and colleagues showed less activation during a working memory task among children age 9–12 with PHIV than controls, and noted a correspondence between areas with less activation and those with decreased cortical thickness [81].

## Conclusions and Current Directions

Several decades of both human and animal research have shown that the developing CNS is vulnerable to HIV and that its effects are distinctive and potentially clinically profound. In recent years, sufficient numbers of young people with PHIV have reached adolescence and young adulthood for a literature regarding neurocognitive and neuroimaging complications during this period of life to develop. The youth participating in this research largely represent those survivors who passed through infancy and early childhood prior to current guidelines recommending cART initiation upon diagnosis of HIV, preferably in early infancy. Many of those now in young adulthood experienced monotherapy, resistance to some medications, and second line therapies during development. Thus, the findings of these studies do not represent the ideal scenario for CNS development of children with PHIV; however, sadly, this ideal scenario often does not occur even today for many children, with significant impairment and encephalopathy remaining issues in lower resource settings [16]. This gives these studies an unfortunate ongoing clinical and public health relevance.

In general, the results of recent studies involving adolescents and young adults with PHIV are consistent with previous observations of an enduring impact on CNS structure and functioning among those with past significant disease progression. This impact is apparent, particularly in specific cognitive domains and brain systems, even following periods of good viral control and extending into adulthood. There is greater variability across studies regarding youth without Class C diagnoses, with some showing differences from PHEU or unexposed youth and others finding that they are comparable once potential sociodemographic influences are taken into account. As longitudinal analyses of maturation across adolescence and into young adulthood have appeared, they have generally shown similar trajectories of development among youth with and without PHIV [44]. However, observed differences in executive function and processing speed change, differing age associations that could relate to developmental cortical pruning, and emerging findings of possible age acceleration illustrate the importance of continued longitudinal studies of CNS maturation among youth with PHIV and raise concerns about very long-term risks to brain health in later adulthood [49, 52•, 77, 78, 82].

As shown in previous studies of children and younger adolescents, this literature suggests optimism in that the majority of youth with PHIV do not have severe impairments [9] and display considerable resilience [83]. However, they remain at risk by virtue not only of their HIV infection but also numerous other environmental or psychosocial stressors that can negatively impact neurodevelopment, such as poverty, food insecurity, trauma, loss of caregivers, caregiver physical and mental illness, barriers to medical care, and stigma and isolation, emphasizing the need to address and measure social and structural determinants of health in research on PHIV [10, 21, 84]. Furthermore, lower neurocognitive functioning has been associated with poorer daily and academic functioning [30–32]. Of concern for older youth, those areas of relative difficulty that are observed, such as executive functioning, processing speed and learning, may present particular risk during the crucial adolescent period where youth experience transition to adult independence and responsibility, age-typical tendencies towards substance use and sexual risk behaviors, and acquisition of critical healthcare self-management skills [36, 85]. In addition, youth with PHIV have high rates of mental health problems and substance use disorders [34, 85–87] which often emerge during adolescence and may impact cognition directly [88] or indirectly through medication adherence [89]. Substance use is a high priority area of research due to its interactions with HIV in affecting CNS functioning and neuropathogenesis through dopamine systems and inflammation, as well as discussion of therapeutic versus harmful effects of cannabis [90–100]. Studies specifically focused on youth are needed due to their different profile of substance use and the potential for greater impact due to exposure during brain development, which continues into young adulthood.

The prevention and treatment of CNS sequelae among youth require a multifaceted approach. The most important strategy currently known for prevention of PHIV impacts on the CNS is early and effective antiretroviral therapy. Thus, the goal of ensuring the best youth outcomes requires global efforts to provide early ART for those children who are born with HIV. Because of the sensitivity of the developing CNS, studies on optimization of ART to balance effectiveness with neurotoxicity and address issues such as penetrance of medications through the blood-brain barrier are critical [9, 10, 12], as is development of pharmacological treatments targeted towards neuropathogenesis and underlying immune and inflammatory mechanisms [101]. The ultimate therapeutic goal, eradicating HIV from the body, requires eliminating the HIV reservoir present in the CNS [102]; cure strategies involving viral activation are complicated in infants and children by the increased vulnerability of their CNS to viral activity. Careful assessment of effects on CNS functioning should be an integral part of cure studies.

A second class of prevention strategies is through addressing the multiple other neurodevelopmental risks faced by children and adolescents with PHIV outlined above. These include structural change to reduce poverty, trauma and adversity, food insecurity, air pollution, systemic inequality and racism, educational and health inequity, and other risks to which children with PHIV are disproportionately exposed [103–105]. Comprehensive care to address mental health and substance use is important and behavioral interventions have been developed for this population [83, 106–108]. This is particularly critical given the aforementioned concerns about developmental effects of substance use during adolescence and complex interactions with cannabis, popular among adolescents [91, 96, 109]. Scalable preventive interventions to boost child outcomes through such strategies as caregiver training are being developed and implemented [10, 103]. Where deficits are present, specialized academic and cognitive remediation programs may help youth optimize their long-term healthcare management, occupational attainment, and quality of life [10, 110–113].

Both research and clinical care regarding long-term CNS problems among those with PHIV rely on measures sensitive to neurocognitive impacts of HIV and treatment that may be subtle. Cognitive and neuroimaging measures should be able to distinguish influences of other developmental risks as well as HIV, reflect maturational processes specific to adolescence and young adulthood, and be appropriate to use longitudinally, minimizing or controlling for repeated measurement effects. Most studies in both the pediatric and adult HIV literatures have used standardized measures of developmental and neuropsychological functioning, sometimes examining their contextual validity or developing local normative data and more commonly by using matched comparison groups [114]. Increasingly, alternative assessment methods are being recommended and/or developed for this population, including locally validated screening measures [9, 115]; computerized, and in some cases portable tablet-based, assessments [78, 116, 117]; exploration of diagnostic classification and criteria [118, 119]; and the use of dynamic assessment or instruments aligned with underlying brain and behavioral processes [10, 120]. Methods that have been developed or tested in the low- to middle-income contexts where pediatric HIV infection is concentrated are particularly critical. At the same time, promising advances in neuroimaging of HIV offer additional tools for research with this population [90].

Ultimately, prevention of the CNS complications of PHIV is best achieved through prevention of PHIV itself. As the world works towards achieving that goal, strategies to maximize adult outcomes for children now being born with PHIV, and those youth living with its effects, will require the commitment of significant resources towards research, prevention, and intervention [4].

## References

[1] UNICEF. Global and regional trends. 2020.

[2] UNAIDS. Fact Sheet 2021: Global HIV Statistics. 2021.

[3] Slogrove AL, Sohn AH (2018). The global epidemiology of adolescents living with HIV: time for more granular data to improve adolescent health outcomes. Curr Opin HIV AIDS.

[4] Vreeman RC, Rakhmanina NY, Nyandiko WM, Puthanakit T, Kantor R (2021). Are we there yet? 40 years of successes and challenges for children and adolescents living with HIV. J Int AIDS Soc.

[5] Smith R, Wilkins M (2015). Perinatally acquired HIV infection: long-term neuropsychological consequences and challenges ahead. Child Neuropsychol.

[6] Sillman B, Woldstad C, McMillan J, Gendelman HE (2018). Neuropathogenesis of human immunodeficiency virus infection. Handb Clin Neurol.

[7] Spudich S, Peterson J, Fuchs D, Price RW, Gisslen M (2019). Potential for early antiretroviral therapy to reduce central nervous system HIV-1 persistence. AIDS (London, England)..

[8] Rowe K, Buivydaite R, Heinsohn T, Rahimzadeh M, Wagner RG, Scerif G, et al. Executive function in HIV-affected children and adolescents: a systematic review and meta-analyses. AIDS Care. 2021:1–25 10.1080/09540121.2021.1873232. 10.1080/09540121.2021.1873232 33764813

[9] Bartlett AW, Williams PCM, Jantarabenjakul W, Kerr SJ (2020). State of the mind: growing up with HIV. Paediatr Drugs..

[10] Benki-Nugent S, Boivin MJ (2019). Neurocognitive complications of pediatric HIV infections. Curr Top Behav Neurosci.

[11] Phillips N, Amos T, Kuo C, Hoare J, Ipser J, Thomas KG, et al. HIV-Associated cognitive impairment in perinatally infected children: a meta-analysis. Pediatrics. 2016;138(5) 10.1542/peds.2016-0893. 10.1542/peds.2016-0893 PMC5079077 27940772

[12] Wilmshurst JM, Hammond CK, Donald K, Hoare J, Cohen K, Eley B (2018). NeuroAIDS in children. Handb Clin Neurol.

[13] Van den Hof M, Ter Haar AM, Caan MWA, Spijker R, van der Lee JH, Pajkrt D (2019). Brain structure of perinatally HIV-infected patients on long-term treatment: a systematic review. Neurol Clin Pract.

[14] Musielak K, Fine J (2016). An updated systematic review of neuroimaging studies of children and adolescent with perinatally acquired HIV. J Pediatr Neuropsychol.

[15] Martín-Bejarano M, Ruiz-Saez B, Martinez-de-Aragón A, Melero H, Zamora B, Malpica NA (2021). A systematic review of magnetic resonance imaging studies in perinatally HIV-infected individuals. AIDS Rev.

[16] Thakur KT, Boubour A, Saylor D, Das M, Bearden DR, Birbeck GL (2019). Global HIV neurology: a comprehensive review. AIDS (London, England).

[17] Ezeamama AE, Zalwango SK, Sikorskii A, Tuke R, Musoke PM, Giordani B (2021). In utero and peripartum antiretroviral exposure as predictor of cognition in 6- to 10-year-old HIV-exposed Ugandan children - a prospective cohort study. HIV Med.

[18] Kerr SJ, Puthanakit T, Vibol U, Aurpibul L, Vonthanak S, Kosalaraksa P (2014). Neurodevelopmental outcomes in HIV-exposed-uninfected children versus those not exposed to HIV. AIDS Care.

[19] McHenry MS, Balogun KA, McDonald BC, Vreeman RC, Whipple EC, Serghides L (2019). In utero exposure to HIV and/or antiretroviral therapy: a systematic review of preclinical and clinical evidence of cognitive outcomes. J Int AIDS Soc.

[20] Centers for Disease Control. Revised classification system for human immunodeficiency virus infection in children less than 13 years of age. MMWR Morb Mortal Wkly Rep. 1994;43:1–12.

[21] Laughton B, Cornell M, Boivin M, Van Rie A (2013). Neurodevelopment in perinatally HIV-infected children: a concern for adolescence. J Int AIDS Soc.

[22] Smith R, Chernoff M, Williams PL, Malee KM, Sirois PA, Kammerer B (2012). Impact of HIV severity on cognitive and adaptive functioning during childhood and adolescence. Pediatr Infect Dis J.

[23] Crowell CS, Huo Y, Tassiopoulos K, Malee KM, Yogev R, Hazra R (2015). Early viral suppression improves neurocognitive outcomes in HIV-infected children. AIDS (London, England).

[24] Jantarabenjakul W, Chonchaiya W, Puthanakit T, Theerawit T, Payapanon J, Sophonphan J (2019). Low risk of neurodevelopmental impairment among perinatally acquired HIV-infected preschool children who received early antiretroviral treatment in Thailand. J Int AIDS Soc.

[25] Laughton B, Cornell M, Grove D, Kidd M, Springer PE, Dobbels E (2012). Early antiretroviral therapy improves neurodevelopmental outcomes in infants. AIDS (London, England).

[26] Panel on Antiretroviral Therapy and Medical Management of Children Living with HIV. Guidelines for the Use of Antiretroviral Agents in Pediatric HIV Infection. Guidelines for the Use of Antiretroviral Agents in Pediatric HIV Infection. Available at https://clinicalinfo.hiv.gov/sites/default/files/guidelines/documents/PediatricGuidelines.pdf. Accessed 17 Jan 2022.

[27] Slogrove AL, Schomaker M, Davies MA, Williams P, Balkan S, Ben-Farhat J (2018). The epidemiology of adolescents living with perinatally acquired HIV: a cross-region global cohort analysis. PLoS Med.

[28] Frigati LJ, Brown K, Mahtab S, Githinji L, Gray D, Zühlke L (2019). Multisystem impairment in South African adolescents with Perinatally acquired HIV on antiretroviral therapy (ART). J Int AIDS Soc.

[29] Flynn PM, Abrams EJ (2019). Growing up with perinatal HIV. AIDS (London, England).

[30] Sirois PA, Chernoff MC, Malee KM, Garvie PA, Harris LL, Williams PL (2016). Associations of memory and executive functioning with academic and adaptive functioning among youth with perinatal HIV exposure and/or infection. J Pediatr Infect Dis Soc.

[31] Phillips N, Thomas KGF, Mtukushe B, Myer L, Zar HJ, Stein DJ, et al. Youth perinatal HIV-associated neurocognitive disorders: association with functional impairment. AIDS Care. 2021:1–5 10.1080/09540121.2021.1891191. 10.1080/09540121.2021.1891191 33625933

[32] Garvie PA, Zeldow B, Malee K, Nichols SL, Smith RA, Wilkins ML (2014). Discordance of cognitive and academic achievement outcomes in youth with perinatal HIV exposure. Pediatr Infect Dis J.

[33] Toska E, Cluver L, Orkin M, Bains A, Sherr L, Berezin M (2019). Screening and supporting through schools: educational experiences and needs of adolescents living with HIV in a South African cohort. BMC Public Health.

[34] Mellins CA, Malee KM (2013). Understanding the mental health of youth living with perinatal HIV infection: lessons learned and current challenges. J Int AIDS Soc.

[35] Nichols SL, Brummel S, Malee KM, Mellins CA, Moscicki AB, Smith R (2021). The role of behavioral and neurocognitive functioning in substance use among youth with perinatally acquired HIV infection and perinatal HIV exposure without infection. AIDS Behav.

[36] Yusuf H, Agwu A (2021). Adolescents and young adults with early acquired HIV infection in the united states: unique challenges in treatment and secondary prevention. Exp Rev Anti-Infect Ther.

[37] UNAIDS. Global AIDS update – Seizing the moment – Tackling entrenched inequalities to end epidemics. 2020. Available at https://www.unaids.org/en/resources/documents/2020/global-aids-report. Accessed 17 Jan 2022.

[38] World Health Organisation. Adolescent health. Available at https://www.who.int/health-topics/adolescent-health#tab=tab_1. Accessed 17 Jan 2022.

[39] Ananworanich J, Kerr SJ, Jaimulwong T, Vibol U, Hansudewechakul R, Kosalaraksa P (2015). Soluble CD163 and monocyte populations in response to antiretroviral therapy and in relationship with neuropsychological testing among HIV-infected children. J Virus Eradication.

[40] Puthanakit T, Ananworanich J, Vonthanak S, Kosalaraksa P, Hansudewechakul R, van der Lugt J (2013). Cognitive function and neurodevelopmental outcomes in HIV-infected children older than 1 year of age randomized to early versus deferred antiretroviral therapy: the PREDICT neurodevelopmental study. Pediatr Infect Dis J.

[41] Tassiopoulos K, Patel K, Alperen J, Kacanek D, Ellis A, Berman C (2016). Following young people with perinatal HIV infection from adolescence into adulthood: the protocol for PHACS AMP Up, a prospective cohort study. BMJ Open.

[42] • Kerr SJ, Puthanakit T, Malee KM, Thongpibul K, Ly PS, Sophonphan J, et al. Increased risk of executive function and emotional behavioral problems among virologically well-controlled perinatally HIV-infected adolescents in Thailand and Cambodia. J Acquir Immune Defic Syndr. 2019;82(3):297–304. 10.1097/qai.0000000000002132. **This study is important in describing cognitive functioning among a cohort of youth with relatively well controlled HIV during development.** 10.1097/QAI.0000000000002132 PMC6814288 31335589

[43] Patel PB, Belden A, Handoko R, Puthanakit T, Kerr S, Kosalaraksa P (2021). Behavioral impairment and cognition in Thai adolescents affected by HIV. Glob Mental Health (Cambridge, England).

[44] Malee KM, Chernoff MC, Sirois PA, Williams PL, Garvie PA, Kammerer BL (2017). Impact of perinatally acquired HIV disease upon longitudinal changes in memory and executive functioning. J Acquir Immune Defic Syndr (1999).

[45] Nichols SL, Chernoff MC, Malee K, Sirois PA, Williams PL, Figueroa V (2016). Learning and memory in children and adolescents with perinatal HIV infection and perinatal HIV exposure. Pediatr Infect Dis J.

[46] Harris LL, Chernoff MC, Nichols SL, Williams PL, Garvie PA, Yildirim C (2018). Prospective memory in youth with perinatally-acquired HIV infection. Child Neuropsychol.

[47] Nichols SL, Chernoff MC, Malee KM, Sirois PA, Woods SP, Williams PL (2016). Executive functioning in children and adolescents with perinatal HIV infection and perinatal HIV exposure. J Pediatr Infect Dis Soc.

[48] Nichols SL, Brummel SS, Smith RA, Garvie PA, Hunter SJ, Malee KM (2015). Executive functioning in children and adolescents with perinatal HIV infection. Pediatr Infect Dis J.

[49] Van den Hof M, Ter Haar AM, Scherpbier HJ, van der Lee JH, Reiss P, Wit F (2020). Neurocognitive development in perinatally human immunodeficiency virus-infected adolescents on long-term treatment, compared to healthy matched controls: a longitudinal study. Clin Infect Dis.

[50] Patel PB, Apornpong T, Puthanakit T, Thongpibul K, Kosalaraksa P, Hansudewechakul R (2019). Trajectory analysis of cognitive outcomes in children with perinatal HIV. Pediatr Infect Dis J.

[51] Judd A, Le Prevost M, Melvin D, Arenas-Pinto A, Parrott F, Winston A (2016). Cognitive function in young persons with and without perinatal HIV in the AALPHI cohort in England: role of non-HIV-related factors. Clin Infect Dis.

[52] • Robbins RN, Zimmerman R, Korich R, Raymond J, Dolezal C, Choi CJ, et al. Longitudinal trajectories of neurocognitive test performance among individuals with perinatal HIV-infection and -exposure: adolescence through young adulthood. AIDS Care. 2020;32(1):21–9. 10.1080/09540121.2019.1626343. **This study highlights processing speed as a potential area of risk over time for youth with PHIV.** 10.1080/09540121.2019.1626343 PMC6883154 31174426

[53] Willen EJ, Cuadra A, Arheart KL, Post MJ, Govind V (2017). Young adults perinatally infected with HIV perform more poorly on measures of executive functioning and motor speed than ethnically matched healthy controls. AIDS Care.

[54] García-Navarro C, Jimenez de Ory S, Velo Higueras C, Zamora B, Prieto L, Ramos JT, et al. Significant differences between verbal and non-verbal intellectual scales on a perinatally HIV-infected cohort: from pediatrics to young adults. Heliyon. 2020;6(4):e03600 10.1016/j.heliyon.2020.e03600. 10.1016/j.heliyon.2020.e03600 PMC7184518 32368635

[55] Coutifaris P, Byrd D, Childs J, Clark U, Posada R, Robbins R (2020). Neurobehavioral outcomes in young adults with perinatally acquired HIV. AIDS (London, England).

[56] Obregon-Perko V, Bricker K, Chahroudi A (2020). The brain retains: nonhuman primate models for pediatric HIV-1 in the CNS. Curr HIV/AIDS Rep.

[57] Hoare J, Ransford GL, Phillips N, Amos T, Donald K, Stein DJ (2014). Systematic review of neuroimaging studies in vertically transmitted HIV positive children and adolescents. Metab Brain Dis.

[58] Li J, Gao L, Wen Z, Zhang J, Wang P, Tu N (2018). Structural covariance of gray matter volume in HIV vertically infected adolescents. Sci Rep.

[59] Li J, Gao L, Ye Z (2021). Study of brain structure in HIV vertically infected adolescents. AIDS Res Hum Retrovir.

[60] Yu X, Gao L, Wang H, Yin Z, Fang J, Chen J (2019). Neuroanatomical changes underlying vertical HIV infection in adolescents. Front Immunol.

[61] Sarma MK, Keller MA, Macey PM, Michalik DE, Hayes J, Nielsen-Saines K (2019). White matter microstructure among perinatally HIV-infected youth: a diffusion tensor imaging study. J Neurovirol.

[62] Ruiz-Saez B, García MM, de Aragon AM, Gil-Correa M, Melero H, Malpica NA (2021). Effects of perinatal HIV-infection on the cortical thickness and subcortical gray matter volumes in young adulthood. Medicine.

[63] Lewis-de Los Angeles CP, Alpert KI, Williams PL, Malee K, Huo Y, Csernansky JG, et al. Deformed subcortical structures are related to past HIV disease severity in youth with perinatally acquired HIV infection. J Pediatr Infect Dis Soc. 2016;5(suppl 1):S6–s14 10.1093/jpids/piw051. 10.1093/jpids/piw051 PMC5181545 27856671

[64] Lewis-de Los Angeles CP, Williams PL, Huo Y, Wang SD, Uban KA, Herting MM, et al. Lower total and regional grey matter brain volumes in youth with perinatally-acquired HIV infection: associations with HIV disease severity, substance use, and cognition. Brain Behav Immun. 2017;62:100–109 10.1016/j.bbi.2017.01.004. 10.1016/j.bbi.2017.01.004 PMC5373952 28089557

[65] • Lewis-de Los Angeles CP, Williams PL, Jenkins LM, Huo Y, Malee K, Alpert KI, et al. Brain morphometric differences in youth with and without perinatally-acquired HIV: a cross-sectional study. NeuroImage Clin. 2020;26:102246 10.1016/j.nicl.2020.102246. **This study presents intriguing findings suggesting possible alterations in maturational cortical pruning in the context of PHIV.** 10.1016/j.nicl.2020.102246 PMC7132093 32251906

[66] Hoare J, Myer L, Heany S, Fouche JP, Phillips N, Zar HJ, et al. Cognition, structural brain changes, and systemic inflammation in adolescents living with HIV on antiretroviral therapy. J Acquir Immune Defic Syndr (1999). 2020;84(1):114–121 10.1097/qai.0000000000002314. 10.1097/QAI.0000000000002314 PMC7141763 32032303

[67] Nwosu EC, Robertson FC, Holmes MJ, Cotton MF, Dobbels E, Little F (2018). Altered brain morphometry in 7-year old HIV-infected children on early ART. Metab Brain Dis.

[68] Casagrande CC, Lew BJ, Taylor BK, Schantell M, O’Neill J, May PE, et al. Impact of HIV-infection on human somatosensory processing, spontaneous cortical activity, and cortical thickness: a multimodal neuroimaging approach. Hum Brain Mapp. 2021;42(9):2851–61. 10.1002/hbm.25408. 10.1002/hbm.25408 PMC8127147 33738895

[69] Kapetanovic S, Norato G, Nair G, Julnes PS, Traino KA, Geannopoulos K, et al. Effect of HIV and interpersonal trauma on cortical thickness, cognition, and daily functioning. J Acquir Immune Defic Syndr (1999). 2020;84(4):405–413 10.1097/qai.0000000000002358. 10.1097/QAI.0000000000002358 PMC8376232 32235173

[70] MacDuffie KE, Brown GG, McKenna BS, Liu TT, Meloy MJ, Tawa B (2018). Effects of HIV Infection, methamphetamine dependence and age on cortical thickness, area and volume. NeuroImage Clin.

[71] Williams ME, Joska JA, Amod AR, Paul RH, Stein DJ, Ipser JC (2020). The association of peripheral immune markers with brain cortical thickness and surface area in South African people living with HIV. J Neurovirol.

[72] Zhao J, Ma Z, Chen F, Li L, Ren M, Li A (2021). Human immune deficiency virus-related structural alterations in the brain are dependent on age. Hum Brain Mapp.

[73] Dean O, Buda A, Adams HR, Mwanza-Kabaghe S, Potchen MJ, Mbewe EG (2020). Brain magnetic resonance imaging findings associated with cognitive impairment in children and adolescents with human immunodeficiency virus in Zambia. Pediatr Neurol.

[74] Wade BSC, Valcour VG, Puthanakit T, Saremi A, Gutman BA, Nir TM (2019). Mapping abnormal subcortical neurodevelopment in a cohort of Thai children with HIV. NeuroImage Clin.

[75] Van den Hof M, Jellema PEJ, Haar AMT, Scherpbier HJ, Schrantee A, Kaiser A, et al. Normal structural brain development in adolescents treated for perinatally acquired HIV: a longitudinal imaging study. AIDS (London, England). 2021 10.1097/qad.0000000000002873. 10.1097/QAD.0000000000002873 PMC8183487 33710018

[76] • Hoare J, Stein DJ, Heany SJ, Fouche JP, Phillips N, Er S, et al. Accelerated epigenetic aging in adolescents living with HIV is associated with altered development of brain structures. J Neurovirol. 2021. 10.1007/s13365-021-00947-3. **This recent neuroimaging study of young adolescents with PHIV raises the issue of the impact of epigenetic aging on brain development in this cohort.** 10.1007/s13365-021-00947-3 PMC8346577 33554325

[77] Horvath S, Stein DJ, Phillips N, Heany SJ, Kobor MS, Lin DTS (2018). Perinatally acquired HIV infection accelerates epigenetic aging in South African adolescents. AIDS (London, England).

[78] Shiau S, Cantos A, Ramon CV, Shen Y, Shah J, Jang G, et al. Epigenetic age in young African American adults with perinatally-acquired HIV. J Acquir Immune Defic Syndr (1999). 2021 10.1097/qai.0000000000002687. 10.1097/QAI.0000000000002687 PMC8217147 33765682

[79] Sarma MK, Pal A, Keller MA, Welikson T, Ventura J, Michalik DE (2021). White matter of perinatally HIV infected older youths shows low frequency fluctuations that may reflect glial cycling. Sci Rep.

[80] Wang P, Li J, Wang X, Thapa D, Wu GY. Asymptomatic human immunodeficiency virus vertical transmitted adolescents’ brain functional changes: based on resting-state functional magnetic resonance imaging. AIDS Res Hum Retrovir. 2018;34(8):699–704. 10.1089/aid.2017.0267. 10.1089/AID.2017.0267 29737186

[81] Heany SJ, Phillips N, Brooks S, Fouche JP, Myer L, Zar H (2020). Neural correlates of maintenance working memory, as well as relevant structural qualities, are associated with earlier antiretroviral treatment initiation in vertically transmitted HIV. J Neurovirol.

[82] Chiappini E, Bianconi M, Dalzini A, Petrara MR, Galli L, Giaquinto C (2018). Accelerated aging in perinatally HIV-infected children: clinical manifestations and pathogenetic mechanisms. Aging.

[83] Malee KM, Smith RA, Mellins CA. Brain and cognitive development among U.S. youth with perinatally acquired human immunodeficiency virus infection. J Pediatr Infect Dis Soc. 2016;5(suppl 1):S1–s5 10.1093/jpids/piw041. 10.1093/jpids/piw041 PMC5181541 27856670

[84] National Institutes of Health Office of AIDS Research. Reduce the incidence of HIV. https://www.oar.nih.gov/hiv-policy-and-research/research-priorities-overview/reduce-incidence-hiv. Accessed 17 Jan 2022.

[85] Vreeman RC, McCoy BM, Lee S (2017). Mental health challenges among adolescents living with HIV. J Int AIDS Soc.

[86] Smith R, Huo Y, Tassiopoulos K, Rutstein R, Kapetanovic S, Mellins C (2019). Mental health diagnoses, symptoms, and service utilization in US youth with perinatal HIV infection or HIV exposure. AIDS Patient Care STDs.

[87] Abrams EJ, Mellins CA, Bucek A, Dolezal C, Raymond J, Wiznia A, et al. Behavioral health and adult milestones in young adults with perinatal HIV infection or exposure. Pediatrics. 2018;142(3) 10.1542/peds.2018-0938. 10.1542/peds.2018-0938 PMC6317560 30097528

[88] Molinaro M, Adams HR, Mwanza-Kabaghe S, Mbewe EG, Kabundula PP, Mweemba M (2021). Evaluating the relationship between depression and cognitive function among children and adolescents with HIV in Zambia. AIDS Behav.

[89] Bucek A, Leu CS, Benson S, Warne P, Abrams EJ, Elkington KS (2018). Psychiatric disorders, antiretroviral medication adherence and viremia in a cohort of perinatally HIV-infected adolescents and young adults. Pediatr Infect Dis J.

[90] Boerwinkle AH, Meeker KL, Luckett P, Ances BM (2021). Neuroimaging the neuropathogenesis of HIV. Curr HIV/AIDS Rep.

[91] Towe SL, Meade CS, Cloak CC, Bell RP, Baptiste J, Chang L (2020). Reciprocal influences of HIV and cannabinoids on the brain and cognitive function. J Neuroimmune Pharmacol.

[92] Saloner R, Fields JA, Marcondes MCG, Iudicello JE, von Känel S, Cherner M (2020). Methamphetamine and cannabis: a tale of two drugs and their effects on HIV, brain, and behavior. J Neuroimmune Pharmacol.

[93] Lin Y, He JJ, Sorensen R, Chang L (2020). Unraveling neuroHIV in the presence of substance use disorders. J Neuroimmune Pharmacol.

[94] Nickoloff-Bybel EA, Calderon TM, Gaskill PJ, Berman JW (2020). HIV Neuropathogenesis in the presence of a disrupted dopamine system. J Neuroimmune Pharmacol.

[95] Kallianpur KJ, Birn R, Ndhlovu LC, Souza SA, Mitchell B, Paul R (2020). Impact of cannabis use on brain structure and function in suppressed HIV infection. J Behav Brain Sci.

[96] Watson CW, Paolillo EW, Morgan EE, Umlauf A, Sundermann EE, Ellis RJ, et al. Cannabis exposure is associated with a lower likelihood of neurocognitive impairment in people living with HIV. J Acquir Immune Defic Syndr (1999). 2020;83(1):56–64 10.1097/qai.0000000000002211. 10.1097/QAI.0000000000002211 PMC6901104 31809361

[97] Hall SA, Lalee Z, Bell RP, Towe SL, Meade CS (2021). Synergistic effects of HIV and marijuana use on functional brain network organization. Prog Neuro-Psychopharmacol Biol Psychiatry.

[98] Skalski LM, Towe SL, Sikkema KJ, Meade CS (2018). Memory impairment in HIV-infected individuals with early and late initiation of regular marijuana use. AIDS Behav.

[99] Skalski LM, Towe SL, Sikkema KJ, Meade CS (2016). The impact of marijuana use on memory in HIV-infected patients: a comprehensive review of the HIV and marijuana literatures. Curr Drug Abuse Rev.

[100] Thames AD, Kuhn TP, Williamson TJ, Jones JD, Mahmood Z, Hammond A (2017). Marijuana effects on changes in brain structure and cognitive function among HIV+ and HIV- adults. Drug Alcohol Depend.

[101] Kapoor A, Tan CS (2020). Immunotherapeutics to Treat HIV in the central nervous system. Curr HIV/AIDS Rep.

[102] Chahroudi A, Wagner TA, Persaud D (2018). CNS persistence of HIV-1 in children: the untapped reservoir. Curr HIV/AIDS Rep.

[103] Boivin MJ, Ruiseñor-Escudero H, Familiar-Lopez I (2016). CNS impact of perinatal HIV infection and early treatment: the need for behavioral rehabilitative interventions along with medical treatment and care. Curr HIV/AIDS Rep.

[104] Suter MK, Karr CJ, John-Stewart GC, Gómez LA, Moraa H, Nyatika D, et al. Implications of combined exposure to household air pollution and HIV on neurocognition in children. Int J Environ Res Public Health. 2018;15(1) 10.3390/ijerph15010163. 10.3390/ijerph15010163 PMC5800262 29361707

[105] Bather JR, Williams PL, Broadwell C, Smith R, Patel K, Garvie PA, et al. Racial/ethnic disparities in longitudinal emotional-behavioral functioning among youth born to women living with HIV. J Acquir Immune Defic Syndr. 2021;87(3):889–898 10.1097/qai.0000000000002665. 10.1097/QAI.0000000000002665 PMC8192436 33675617

[106] Nestadt DF, Saisaengjan C, McKay MM, Bunupuradah T, Pardo G, Lakhonpon S (2019). CHAMP+ Thailand: pilot randomized control trial of a family-based psychosocial intervention for perinatally HIV-infected early adolescents. AIDS Patient Care STDs.

[107] Bhana A, Mellins CA, Small L, Nestadt DF, Leu CS, Petersen I, et al. Resilience in perinatal HIV+ adolescents in South Africa. AIDS Care. 2016;28 Suppl 2(sup2):49–59 10.1080/09540121.2016.1176676. 10.1080/09540121.2016.1176676 PMC4991226 27391999

[108] McKernan McKay M, Alicea S, Elwyn L, McClain ZR, Parker G, Small LA, et al. The development and implementation of theory-driven programs capable of addressing poverty-impacted children's health, mental health, and prevention needs: CHAMP and CHAMP+, evidence-informed, family-based interventions to address HIV risk and care. J Clin Child Adolesc Psychol 2014;43(3):428–441 10.1080/15374416.2014.893519. 10.1080/15374416.2014.893519 PMC4215567 24787707

[109] Yadav-Samudrala BJ, Fitting S (2021). Mini-review: The therapeutic role of cannabinoids in neuroHIV. Neurosci Lett.

[110] Fraser S, Cockcroft K (2020). Working with memory: computerized, adaptive working memory training for adolescents living with HIV. Child Neuropsychol.

[111] Pennar A, Naar S, Woods S, Nichols S, Outlaw A, Ellis D (2018). Promoting resilience through neurocognitive functioning in youth living with HIV. AIDS Care.

[112] Woods SP, Doyle KL, Morgan EE, Naar-King S, Outlaw AY, Nichols SL (2014). Task importance affects event-based prospective memory performance in adults with HIV-associated neurocognitive disorders and HIV-infected young adults with problematic substance use. J Int Neuropsychol Soc.

[113] Chan T, Marta M, Hawkins C, Rackstraw S (2020). Cognitive and neurologic rehabilitation strategies for central nervous system HIV infection. Curr HIV/AIDS Rep.

[114] Chernoff MC, Laughton B, Ratswana M, Familiar I, Fairlie L, Vhembo T (2018). Validity of neuropsychological testing in young African children affected by HIV. J Pediatr Infect Dis.

[115] Phillips NJ, Thomas KGF, Myer L, Sacktor N, Zar HJ, Stein DJ (2019). Screening for HIV-associated neurocognitive disorders in perinatally infected adolescents: youth-International HIV Dementia Scale validation. AIDS (London, England).

[116] Robbins RN, Kluisza L, Liu J, Santoro AF, Raymond J, Ngyuen N (2020). Construct validity supports use of a novel, tablet-based neurocognitive assessment for adolescents and young adults affected by perinatal HIV from vulnerable communities in the United States. AIDS Behav.

[117] Scott JC, Van Pelt AE, Port AM, Njokweni L, Gur RC, Moore TM (2020). Development of a computerised neurocognitive battery for children and adolescents with HIV in Botswana: study design and protocol for the Ntemoga study. BMJ Open.

[118] Paul RH, Cho KS, Belden AC, Mellins CA, Malee KM, Robbins RN (2020). Machine-learning classification of neurocognitive performance in children with perinatal HIV initiating de novo antiretroviral therapy. AIDS (London, England).

[119] Hoare J, Phillips N, Joska JA, Paul R, Donald KA, Stein DJ (2016). Applying the HIV-associated neurocognitive disorder diagnostic criteria to HIV-infected youth. Neurology..

[120] Sanislow CA, Ferrante M, Pacheco J, Rudorfer MV, Morris SE (2019). Advancing translational research using NIMH research domain criteria and computational methods. Neuron..

